# Behavioral Effects of Repetitive Transcranial Magnetic Stimulation in Disorders of Consciousness: A Systematic Review and Meta-Analysis

**DOI:** 10.3390/brainsci13101362

**Published:** 2023-09-23

**Authors:** Zihan Yang, Tian Yue, Volker R. Zschorlich, Dai Li, Duanwei Wang, Fengxue Qi

**Affiliations:** 1 School of Sports Medicine and Rehabilitation, Beijing Sport University, Beijing 100084, China; 2 Institute of Sport Science, Carl von Ossietzky Universität Oldenburg, 26129 Oldenburg, Germany; 3 Department of Sports Medicine, Peking University Third Hospital, Institute of Sports Medicine of Peking University, Beijing 100191, China; 4 Beijing Key Laboratory of Sports Injuries, Beijing 100191, China; 5 Engineering Research Center of Sports Trauma Treatment Technology and Devices, Ministry of Education, Beijing 100191, China; 6 Shandong Mental Health Center, Shandong University, Jinan 250012, China; 7 Sports, Exercise and Brain Sciences Laboratory, Beijing Sport University, Beijing 100084, China

**Keywords:** repetitive transcranial magnetic stimulation, disorders of consciousness, recovery

## Abstract

Traumatic brain injury, cardiac arrest, intracerebral hemorrhage, and ischemic stroke may cause disorders of consciousness (DoC). Repetitive transcranial magnetic stimulation (rTMS) has been used to promote the recovery of disorders of consciousness (DoC) patients. In this meta-analysis, we examined whether rTMS can relieve DoC patient symptoms. We searched through journal articles indexed in PubMed, the Web of Science, Embase, Scopus, and the Cochrane Library until 20 April 2023. We assessed whether studies used rTMS as an intervention and reported the pre- and post-rTMS coma recovery scale-revised (CRS-R) scores. A total of 207 patients from seven trials were included. rTMS significantly improved the recovery degree of patients; the weighted mean difference (WMD) of the change in the CRS-R score was 1.89 (95% confidence interval (CI): 1.39–2.39; *p* < 0.00001) in comparison with controls. The subgroup analysis showed a significant improvement in CRS-R scores in rTMS over the dorsolateral prefrontal cortex (WMD = 2.24; 95% CI: 1.55–2.92; *p* < 0.00001; *I*^2^ = 31%) and the primary motor cortex (WMD = 1.63; 95% CI: 0.69–2.57; *p* = 0.0007; *I*^2^ = 14%). Twenty-hertz rTMS significantly improved CRS-R scores in patients with DoC (WMD = 1.61; 95% CI: 0.39–2.83; *p* = 0.010; *I*^2^ = 31%). Furthermore, CRS-R scores in rTMS over 20 sessions significantly improved (WMD = 1.75; 95% CI: 0.95–2.55; *p* < 0.0001; *I*^2^ = 12%). rTMS improved the symptoms of DoC patients; however, the available evidence remains limited and inadequate.

## 1. Introduction

Disorders of consciousness (DoC) refers to alternations in arousal or awareness, which are commonly caused by traumatic brain injury, cardiac arrest, intracerebral hemorrhage, and ischemic stroke [1]. The levels of DoC are distinguished using the coma recovery scale-revised (CRS-R) score. Patients with DoC are categorized as being in a coma, vegetative state (VS; known as unresponsive wakefulness syndrome (UWS)), and minimally conscious state (MCS) [2]. Some studies have shown that compared with other behavioral assessment scales, the CRS-R scale is more sensitive than other scales and contains evaluation criteria for DoC patients [3,4,5]. Therefore, in this study, the CRS-R scale was used as a standard for the improvement of the consciousness level in the diagnosis of patients with DoC.

It has been shown that the thalamus-based consciousness system is disrupted in patients with DoC. The functional connectivity between the bilateral thalamus and the whole brain is damaged [6]. A study showed that a lesion of the pontine tegmentum is significantly related to DoC [7]. Two cortical regions between the ventral anterior insula and pregenual anterior cingulate cortex become disconnected in patients with DoC [7]. Regardless of the cause, the physiological mechanism is the widespread extinction of excitatory synaptic activity in the cerebral cortex [8]. The loss of direct structural input or the reduction in neuronal input in the neocortex and thalamus result in a process called “disfacilitation”, which leads to a decrease in the neuronal firing rate [1]. The development of diagnostic and prognostic techniques provides new opportunities to detect consciousness recovery and increase the treatment potential of patients diagnosed with DoC [1]. Some studies have shown that most patients with DoC regained consciousness and functional recovery during rehabilitation, proving that effective rehabilitation intervention methods are meaningful for consciousness improvement in patients with DoC. They also suggested that caution is necessary in deciding whether patients with DoC should discontinue treatment [9,10,11,12]. Nevertheless, the available intervention strategies for facilitating the restoration of consciousness are restricted in scope. Insufficiently recognized are the suitable and efficacious therapies for patients diagnosed with DoC. In recent times, there has been a growing utilization of noninvasive brain stimulation methods to enhance the process of regaining consciousness in individuals who have experienced significant brain injury.

Repetitive transcranial magnetic stimulation (rTMS) is a pain-free and indirect method used to induce excitability changes in the cortex via a wire coil generating a magnetic field. The stimulation frequency of rTMS is divided into high-frequency and low-frequency. Low-frequency stimulation (<1 Hz) has inhibitory effects and induces long-term depression-like plasticity, while high-frequency stimulation (≥5 Hz) causes a brain excitation effect and induces long-term potentiation-like plasticity [13].

The dorsolateral prefrontal cortex (DLPFC) is one of the main target stimulation areas of rTMS [14]. A study indicated that high-frequency rTMS over the DLPFC elicits an enhancement in cortical activity in patients with obsessive–compulsive disorder, but low-frequency rTMS over the DLPFC induced an opposite impact [15]. However, the recovery of consciousness is not just associated with a single brain region. Research has demonstrated that individuals with DoC have disturbances in the brain’s functional networks responsible for processing internal thoughts and external stimuli. These disruptions are observed both during periods of rest and when engaged in goal-oriented tasks, and are found to be distinct from those observed in healthy individuals [16]. One potential etiological factor of DoC could be the disruption of the default-mode network (DMN) [17]. The extent of modified brain functional network connectivity may be correlated with the degree of impaired consciousness and the restoration of connectivity could be linked to the recovery of consciousness [16]. In addition, the primary motor cortex (M1) is another possible helpful target stimulation area of rTMS for the motor rehabilitation of patients with neurological disorders. For example, the M1 on the affected side undergoes substantial alterations and impairs motor function in patients with stroke [18]. The M1 was shown to be suppressed on the afflicted side and overactivated on the unaffected side [19]. In order to alleviate patient symptoms, the excitation of the M1 on the affected side and the inhibition of the M1 on the unaffected side are frequently employed [20,21]. Low-frequency rTMS has not only been shown to effectively decrease the cortical excitability of the unaffected M1, but also increased that of the affected M1 at the same time, while high-frequency rTMS increased the cortical excitability of M1 on the affected side in patients with stroke [22]. Some studies reported that rTMS over the M1 could affect other brain regions and remedy patients with DoC [23,24,25,26]. A recent study showed that rTMS can be used over the posterior parietal cortex (PPC) to treat patients with DoC; however, the effectiveness requires further studies [27].

This meta-analysis aimed to systematically assess the efficacy of (1) rTMS on improving the symptoms of DoC patients and (2) the potential of different rTMS stimulation regions, stimulation frequencies, and stimulus durations on improving the symptoms of patients with DoC using subgroup analyses.

## 2. Materials and Methods

### 2.1. Search Strategy

**Figure 1:**
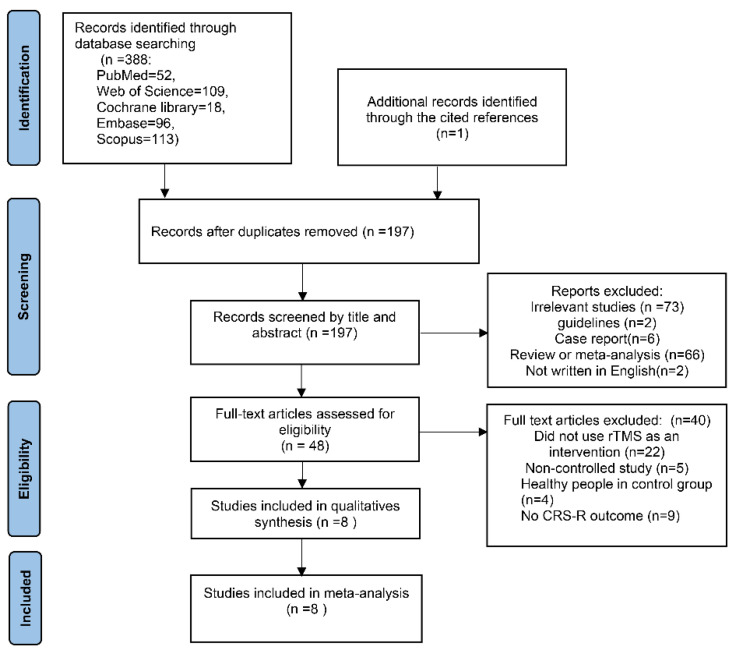
The flowchart of the search procedure. rTMS: repetitive transcranial magnetic stimulation; CRS-R: coma recovery scale-revised.

The Preferred Reporting Items for Systematic Reviews and Meta-Analyses (PRISMA) guidelines were utilized to conduct this systematic review on the effectiveness of utilizing rTMS as an intervention for patients with DoC. We searched through journal articles indexed in PubMed, Embase, Cochrane, Scopus, and the Web of Science until 20 April 2023. To identify studies using rTMS as an intervention for DoC patients, the following search terms were used: “minimally conscious state”, “vegetative state”, “disorder of consciousness”, or “transcranial magnetic stimulation”. Lastly, a thorough search was conducted on the list of references of the articles included in the study to identify any new trials that were pertinent to the research. The search procedure is depicted in Figure 1.

We registered the protocol on the international prospective register of systematic reviews (https://www.crd.york.ac.uk/prospero/ (accessed on 22 August 2023)), for which the registration number is CRD42023453758.

### 2.2. Inclusion and Exclusion Criteria

This meta-analysis included seven randomized controlled trials (RCTs) and one nonrandomized trial that investigated the effectiveness of rTMS in individuals diagnosed with DoC. Due to the limited number of RCTs, the search was expanded to include one nonrandomized trial. The inclusion and exclusion criteria were detailed below.

Studies were included if: (1) patients were diagnosed with MCS or VS/UWS; (2) rTMS was used as an intervention; (3) a sham stimulation was performed as the control group; and (4) the pre- and post-rTMS CRS-R was measured in the DoC patients. Studies were excluded if: (1) they were published as a meta-analysis, review, case report, guideline, or book; (2) no control trials or single-group pre- and post-test experiments were performed; (3) not published in English; and (4) animal trials.

### 2.3. Quality Assessment

**Figure 2:**
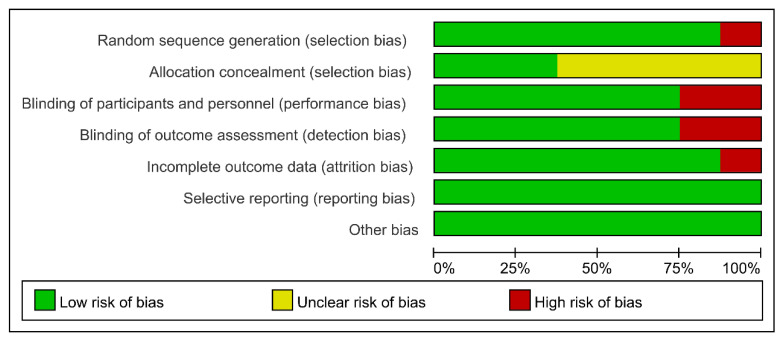
Risk of bias graph.

Two authors assessed the quality of the eight included articles independently. The Cochrane collaboration tool, which has seven domain biases, was utilized. Three levels of bias risk (high, low, and unclear) were applied to grade the included studies. The articles included three high-quality studies with a low-level bias risk [27,28,29], two studies with a high level of bias risk [30,31], and three with a moderate risk of bias [24,25,26]. The risk of bias is shown in Figure 2. Two independent authors rated each study and extracted the information. Any disagreement was resolved through discussion between the two authors.

### 2.4. Data Extraction

The relevant data extracted from each study included: (1) the authors and publication year; (2) the sample size and participant characteristics; (3) details of the stimulation protocol, such as the specific brain target region, stimulation frequency, stimulation duration, and design of the sham stimulation; and (4) the outcome measurements, encompassing behavioral outcomes, if they were provided.

### 2.5. Data Analysis

All results of this meta-analysis were calculated using Review Manager software (Review Manager 5.4). CRS-R was utilized to measure the degree of consciousness. The weighted mean difference (WMD) was employed to integrate the effect size when the outcomes were measured utilizing the same scales. In instances where there was a significant level of heterogeneity or a lack of consistency in the units of weights and measurements, as well as the techniques of measurement, the standardized mean difference (SMD) was employed to integrate the effect size. In addition, the effect sizes of the experimental and sham control groups were synthesized through the changes between the post-intervention values and the baseline. We also obtained the change values (mean ± standard deviation). The value of *I*^2^ less than or equal to 50% suggested a low degree of heterogeneity, indicating that the results should be combined using a fixed-effect model. On the other hand, *I*^2^ values ranging from 50% to 75% and greater than 75% indicated moderate and high levels of heterogeneity, correspondingly [26]. In such cases, the appropriate approach for combining the data would be to use a random effect model. *p* < 0.05 was deemed to indicate statistical significance.

## 3. Results

### 3.1. Search Results

According to the above search strategy, we found 388 articles in the initial search, and 197 articles remained after excluding duplicate articles. Of these, 66 articles were meta-analyses or reviews and 2 articles were not written in the English language. In addition, six case reports and two guidelines were also excluded. After reading the abstracts and titles, articles unrelated to the main content of this meta-analysis were excluded. Finally, if the articles did not conform to the content of this study after reading the complete text, the articles were excluded. A total of eight articles were ultimately included.

### 3.2. Characteristics of Included Studies

The characteristics of the included studies are shown in Table 1. The eight articles included rTMS as the intervention, with the placebo group as the control group. All studies assessed behavioral outcomes using the CRS-R score. A recent study demonstrated that patients with DoC after brain injury may benefit from continuous functional monitoring and new rehabilitation programs for the first decade [9]. Therefore, the participants included after brain injury less than ten years.

### 3.3. Heterogeneity Analysis

To demonstrate the reliability of this study, we excluded the literature one by one, and found that one of the articles was highly heterogeneous. The total heterogeneity analysis reported that the study of Shen et al. had a significant effect on the result of this meta-analysis; the heterogeneity was 67% (*p* = 0.004) [29]. If we removed this article, the total heterogeneity was reduced to 20% (*p* = 0.08), and the heterogeneity of the subgroup was reduced to 0% (*p* = 0.999). In the study of Shen et al., all patients in the experimental group were administered conventional rehabilitation interventions, which consisted of a 20 min session of electrical stimulation targeting the median nerve, followed by a 30 min period of passive limb movement, and concluding with a 40 min hyperbaric oxygen treatment. This might have been the reason for the large heterogeneity. Therefore, to improve the accuracy of this meta-analysis, we excluded this study, and the meta-analysis was performed on the remaining seven studies. In the subgroup of 10 Hz, there was another article with high heterogeneity [31]; the heterogeneity of the subgroup was 79% (*p* = 0.008). If we removed this article, the heterogeneity of the subgroup reduced to 0% (*p* = 0.71); thus, we excluded this study in the subgroup of 10 Hz. Therefore, due to the high heterogeneity of this paper, subgroup analyses could not be carried out. There was only one article in the one-session group, so we did not analyze this subgroup.

### 3.4. Meta-Analysis in All Protocols

**Figure 3:**
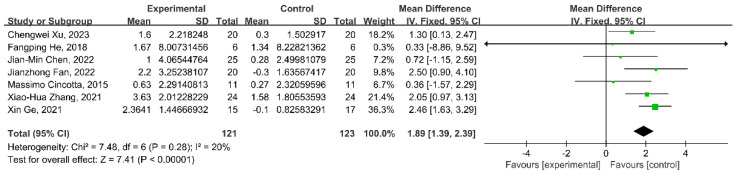
Meta−analysis of all protocols on the change in CRS-R score in patients with disorders of consciousness [24,25,26,27,28,30,31].

The heterogeneity was tested in the seven articles (*I*^2^ = 20%, *p* = 0.28); thus, the meta-analysis could be conducted with a fixed effects model. This meta-analysis demonstrated that the WMD in the change in the CRS-R score was 1.89 (95% confidence interval (CI) 1.39–2.39; *p* < 0.00001) between the rTMS and sham control group (Figure 3).

### 3.5. Subgroup Analysis: Target Region

**Figure 4:**
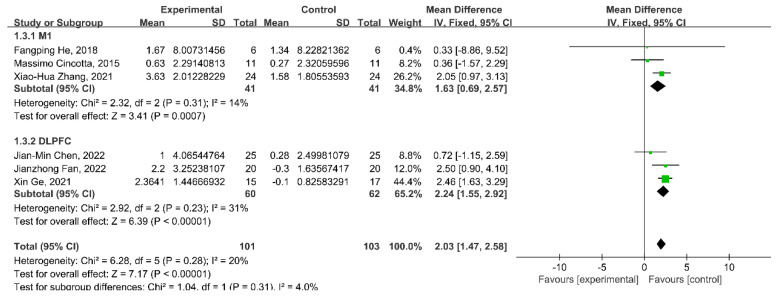
Meta-analysis of rTMS over the M1 and the DLPFC on the change in CRS-R score. M1: primary motor cortex; DLPFC: dorsolateral prefrontal cortex [24,25,26,28,30,31].

This meta-analysis conducted subgroup analyses in different target brain regions. The subgroup analysis showed a significant improvement in the CRS-R scores of rTMS targeting the DLPFC (WMD = 2.24; 95% CI: 1.55–2.92; *p* < 0.00001; *I*^2^ = 31%), as well as the change in CRS-R scores in rTMS targeting the M1 (WMD = 1.63; 95% CI: 0.69–2.57; *p* = 0.0007; *I*^2^ = 14%) (Figure 4).

### 3.6. Subgroup Analysis: Stimulation Frequency

**Figure 5:**
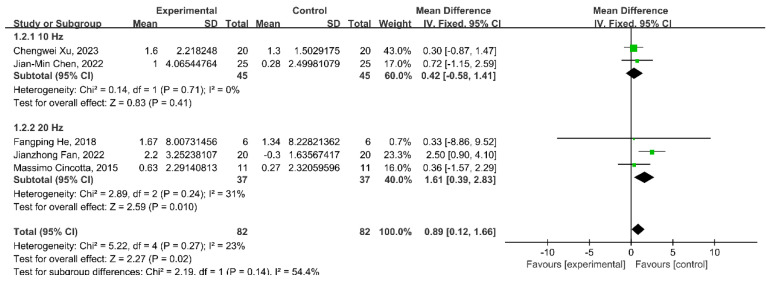
Meta-analysis of rTMS of 10 Hz and 20 Hz on the change in CRS-R score [24,25,27,28,30].

In different stimulation frequencies, the research results demonstrated a statistically significant enhancement in CRS-R scores following the administration of the 20 Hz rTMS intervention (WMD = 1.61; 95% CI: 0.39–2.83; *p* = 0.010; *I*^2^ = 31%); however, there was no significant change in the 10 Hz rTMS intervention (WMD = 0.42; 95% CI: −0.58–1.41; *p* = 0.41; *I*^2^ = 0%) (Figure 5).

### 3.7. Subgroup Analysis: Stimulation Duration

**Figure 6:**
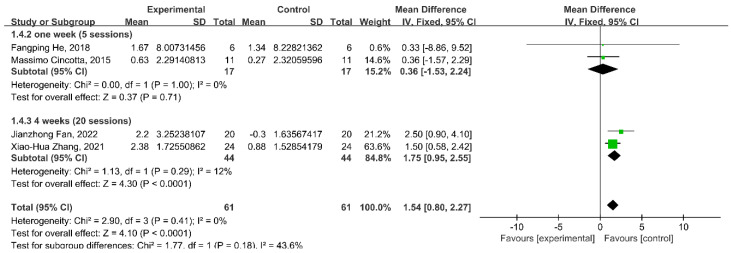
Meta-analysis of different stimulation duration on the change in CRS-R score [24,25,26,28].

In different stimulation durations, the results reported that 20 sessions during 4 weeks of rTMS intervention significantly improved CRS-R scores (WMD = 1.75; 95% CI: 0.95–2.55; *p* < 0.0001; *I*^2^ = 12%); however, there were no significant changes in the CRS-R scores of five sessions (WMD = 0.36; 95% CI: −1.53–2.24; *p* = 0.71; *I*^2^ = 0%) of the rTMS intervention (Figure 6).

## 4. Discussion

This meta-analysis was performed on seven articles to examine the efficacy of the rTMS intervention, as compared to sham controls, in improving the symptoms of 207 patients with DoC. The results indicated that rTMS improved the recovery of patients with DoC, but different stimulation frequencies, stimulation sessions, and stimulation brain regions produced different effects.

High-frequency rTMS could enhance the expression of brain-derived neurotrophic factors by activating the Ca^2+^ signaling pathway [32]. rTMS induced synaptic plasticity and improved brain functional connectivity to promote the recovery of patients with DoC [33]. A study showed that high- or low-frequency rTMS applied to the M1 increased the cortical excitability on the stimulated side, which would increase the patients’ CRS-R scores and promote the recovery of motor function [22]. The results of our meta-analysis showed that 20 Hz rTMS positively promoted the recovery of DoC patients, but 10 Hz rTMS did not induce significant changes.

The research results additionally demonstrated that 4 weeks of rTMS improved the CRS-R scores among individuals diagnosed with disorders of consciousness, but five stimulation sessions of rTMS did not significantly improve the symptoms of patients with DoC. This could be because the stimulation sessions affected the efficacy in improving the symptoms of patients with DoC because all studies using the 20 Hz rTMS intervention stimulated for more than five sessions. Zhang et al. reported that 40 sessions of rTMS worked better than 20 sessions of rTMS intervention, suggesting that patients with DoC require long-term rTMS therapeutic intervention [26]. It may be that brain plasticity can only be observed after a long period of rTMS treatment [34]. However, there was a lack of studies comparing long-term versus short-term rTMS intervention; therefore, we could not be sure if long-term rTMS was more effective. However, rTMS was suggested to be a viable treatment for patients with DoC.

The results showed that rTMS targeting the DLPFC and M1 improved the symptoms of patients with DoC. The result of this meta-analysis was consistent with Feng et al., who reported that noninvasive brain stimulation over the DLPFC could improve the recovery of consciousness among individuals diagnosed with DoC [35]. Several studies demonstrated that there is a disruption in the functional networks of the brain that are essential for processing internal thoughts and external stimuli in patients diagnosed with DoC [16]. The interruption of the DMN may be a cause of DoC [17]. Some studies reported that the DLPFC is a core hub and rTMS targeting the DLPFC can regulate DMN connectivity [36,37,38]. The M1 is also a target region for improving the symptoms of patients with DoC. However, a study reported that rTMS over the M1 was ineffective [24]. One possible reason is that the cortical connections in patients with DoC are completely or almost completely disordered [1], which may result in the lack of a neural network capable of reacting as an effective matrix to the effects of rTMS applied to the M1 [39]. This hypothesis was consistent with demonstrating the severe functional impairment of brain interregional connectivity in VS tested simultaneously with transcranial magnetic stimulation and electroencephalograph recordings [40]. Therefore, rTMS applied to the M1 may not be the most appropriate target area in patients with DoC; therefore, more studies are needed to prove that rTMS targeting the M1 is an effective stimulation region.

The PPC holds growing significance in the clinical rehabilitation of individuals with DoC. It serves as a vital connection within the DMN and assumes a pivotal role in the recovery process of patients with DoC [41]. The DMN is commonly acknowledged as a collection of cortical regions, such as the left and right middle frontal gyrus, bilateral medial frontal, left and right middle temporal, occipital gyrus, and bilateral precuneus, among other locations [42]. The recovery of individuals with DoC is not only dependent on a single brain region. The integrity of the default-mode network (DMN) has been found to potentially have a correlation with the levels of residual consciousness in individuals diagnosed with disorders of consciousness [43]. Hence, the utilization of the rTMS on PPC may potentially contribute to facilitating the rehabilitation process of individuals diagnosed with DoC. To date, there has been limited research conducted on the impact of rTMS specifically targeting the PPC in ameliorating the symptoms experienced by individuals with DoC. This area of investigation has been explored in a single paper, thus far [27]. The research results of this study showed that the left PPC holds significant potential as a target for rTMS interventions aimed at enhancing functional recovery in patients who have a positive response. The study found a substantial rise in the total score in the CRS-R in the group that received rTMS compared to the group that received a sham treatment. This suggests that applying rTMS over the left PPC can significantly enhance the consciousness in patients diagnosed with DoC. This phenomenon could perhaps be attributed to the left PPC, which is an integral component of the DMN. Enhancing the activity of the left PPC is essential for the restoration of consciousness and has been linked to various etiologies of injury in patients [41].

The research findings indicated that rTMS was associated with a significant increase in the possibility of experiencing minor adverse effects, including headache, pain, dizziness, drowsiness, and dry mouth [44,45,46]. It is important to point out that these symptoms tended to rapidly diminish upon the withdrawal of rTMS [45]. rTMS is generally considered to be a safe and well-tolerated intervention [44]. Hence, rTMS may be consider a safe practice.

In the case of rTMS as an intervention, the investigator must check the patient for a variety of risk factors, including, but not limited to, potential effects on brain function and the occurrence of seizures [47]. In addition, the effects of rTMS on medical devices, such as pacemakers, brain devices, and hearing aids, warrant careful consideration. If the patient is pregnant, it is crucial to analyze the potential consequences of rTMS on the unborn [48]. If a patient is determined to be susceptible to negative outcomes, it becomes necessary to conduct supplementary safety investigations and undertake vigilant safety monitoring throughout the process of clinical trials. When a patient exhibits a heightened susceptibility to experiencing severe adverse effects, it is imperative to conduct a meticulous assessment of the risk–benefit ratio and take precautions in the administration of rTMS [47,48]. The safety of stimulation levels may vary based on the specific cortical region being targeted and the corresponding risk–benefit ratio. Furthermore, the criteria for determining an acceptable level of risk may also differ [47].

This meta-analysis also had some limitations. Firstly, we did not include unpublished studies and could have overlooked relevant research publications in languages other than English. Secondly, due to the limited number of relevant studies, six articles were RCTs and one article was not randomly assigned. Finally, our analysis of the stimulation duration did not include a subgroup due to there only being one study.

It is the hope that future treatment studies explore the impact of various stimulation parameters, such as the stimulation frequency, stimulation duration, and stimulation target region, in order to identify a more suitable dosage of stimulation. Furthermore, it is expected that further research explores the utilization of rTMS as a potential intervention to enhance the level of consciousness among those afflicted with disorders of consciousness.

## 5. Conclusions

This meta-analysis found that the rTMS intervention is safe and effective in improving the symptoms of patients with disorders of consciousness. It was proposed that in order to enhance the effectiveness of rTMS, a frequency of 20 Hz should be delivered to either the DLPFC or the M1 for a total of 20 sessions. This approach aims to facilitate the restoration of consciousness in individuals diagnosed with DoC.

## Figures and Tables

**Table 1:** Characteristics of included studies.

Authors	Study Design	Participants	Time after Injury	Intervention	Duration	Brain Target	Control	Behavioral Outcome
Massimo Cincotta et al., 2015 [24]	Cross-over	11 VS	9–80 months	20 Hz rTMS	10 min per session; 5 sessions	LM1	The utilization of a sham coil in rTMS	CRS-R
Fangping He et al., 2018 [25]	Cross-over	3 VS/UWS, 3 MCS	1–3 months	20 Hz rTMS	10 min per session; 5 sessions	LM1	Sham rTMS by positioning the coil away from the head	CRS-R
Xin Ge et al., 2021 [31]	Parallel	32 VS	<1 month	10 Hz rTMS	20 min per session; 1 session	RDLPFC	The utilization of a sham coil in rTMS	CRS-R
Xiao-Hua Zhang et al., 2021 [26]	Parallel	48 PVS	>3 months	5 Hz rTMS	40 sessions (5 times a week over 8 consecutive weeks)	LM1	Sham rTMS using a sham coil	CRS-R
Jian-Min Chen et al., 2022 [30]	Parallel	50 PVS	1–3 months	10 Hz rTMS	20 min per session; 1 session	LDLPFC (F3)	The utilization of a sham coil in rTMS	CRS-R
Jianzhong Fan et al., 2022 [28]	Parallel	40 PVS	1–3 months	20 Hz rTMS	20 sessions (5 times a week over 4 consecutive weeks)	LDLPFC (F3)	The utilization of a sham coil in rTMS	CRS-R
Chengwei Xu et al., 2023 [27]	Cross-over	20 UWS	<12 months and >28 days	10 Hz rTMS	20 min per session; 10 sessions	LPPC	The utilization of a sham coil in rTMS	CRS-R
Longbin Shen et al., 2023 [29]	Parallel	99 VS	1–3 months	20 Hz rTMS	10 min per session; 5 sessions	LDLPFC (F3)	The utilization of a sham coil in rTMS	CRS-R

VS: vegetative state; UWS: unresponsive wakefulness syndrome; MCS: minimally conscious state; PVS: persistent vegetative state; rTMS: repetitive transcranial magnetic stimulation; LM1: left primary motor cortex; RDLPFC: right dorsolateral prefrontal cortex; LDLPFC: left dorsolateral prefrontal cortex; LPPC: left posterior parietal cortex; CRS-R: coma recovery scale-revised.

## Data Availability

Both the lead author and the corresponding author can gladly provide access to the data utilized in this study upon request.
